# Angiogenic and Inflammatory Properties of Psoriatic Arthritis

**DOI:** 10.1155/2013/630620

**Published:** 2013-05-30

**Authors:** Toshiyuki Yamamoto

**Affiliations:** Department of Dermatology, Fukushima Medical University, Fukushima 960-1295, Japan

## Abstract

Psoriatic arthritis (PsA) is a chronic inflammatory arthropathy associated with psoriasis and included in seronegative spondyloarthropathy. PsA has several unique characteristics different from rheumatoid arthritis (RA), such as enthesopathy, dactylitis, and abnormal bone remodeling. As compared with synovitis of RA (pannus), proliferation of PsA synovium is mild and characterized by hypervascularity and increased infiltration of polymorphonuclear leukocytes in the synovial tissues. Angiogenesis plays a crucial role in cutaneous psoriasis, and several angiogenic factors such as vascular endothelial growth factor, interleukin-8, angiopoietin, tumor necrosis factor-**α** and transforming growth factor-*β*, are suggested to play an important role also in the pathophysiology of PsA. Further, IL-17 has various functions such as upregulation of proinflammatory cytokines, attraction of neutrophils, stimulation of keratinocytes, endothelial cell migration, and osteoclast formation via RANKL from activated synovial fibroblasts. Thus, IL-17 may be important in angiogenesis, fibrogenesis, and osteoclastogenesis in PsA. In this paper, roles of angiogenesis in the psoriatic synovium are discussed, which may strengthen the understanding of the pathogenesis of PsA.

## 1. Introduction

Psoriatic arthritis (PsA) is a chronic inflammatory arthropathy in association with psoriasis. PsA is classified as one of the seronegative spondyloarthropathy characterized by joint destruction with extra-articular involvement (i.e., eye, gut, and bowel). The involved joints present swelling, redness, and deformity to the end, which are usually periphery (main involvements are distal interphalangeal joints), but sacroiliac joints are less frequently affected. In nearly 70% of patients, cutaneous lesions precede the onset of joint pain, in 20% arthropathy starts before skin manifestations, and in 10% both are contemporary. Results of laboratory examination show elevated levels of erythrocyte sedimentation rate (ESR) and C-reactive protein (CRP) which reflect acute-phase inflammation. Rheumatoid factor (RF) and anti-cyclic citrullinated peptide (CCP) antibodies are usually negative. HLA-B27 is found chiefly in patients with spondylitis type PsA. Radiographic features of joint space narrowing, erosions, osteolysis, new bone formation, enthesitis, spur formation, pencil-in-cup appearance, and bamboo spine. Although, in general, patients with severe cutaneous psoriasis often complain of arthralgia, skin severity and joint lesions are not always parallel. A number of studies have suggested genetic, environmental, and immunological aspects in the development of PsA. Although the pathophysiology of synovium of PsA is still not fully elucidated [1, 2], previous studies suggested that the characteristic features of PsA synovium include hypervascularity and increased polymorphonuclear leukocyte infiltrates [3], which resembles that of spondyloarthritis, rather than rheumatoid arthritis (RA) [4]. Angiogenesis is suggested to play an important role in the early event in PsA. Herein, angiogenic properties in cutaneous and joint lesions of PsA are discussed.

## 2. Angiogenesis of Cutaneous Psoriasis

A number of studies have demonstrated that angiogenesis is crucial in the pathogenesis of psoriasis [5–7]. Early lesion of psoriasis is histologically characterized by capillary dilation and edema in the papillary dermis with perivascular lymphocytic infiltration, prior to the onset of epidermal proliferation. On the other hand, proangiogenic environment is induced by helper T-cells and regulatory T-cells. Development of psoriasis is in accordance with the elevated levels of vascular endothelial growth factor (VEGF) in the plasma and skin. Psoriatic keratinocytes produce several angiogenic cytokines, such as VEGF, interleukin-8 (IL-8), tumor necrosis factor-*α* (TNF-*α*), and transforming growth factor-β (TGF-β). Additionally, infiltrating neutrophils can be a source of VEGF, which induces neutrophil chemotaxis in an autocrine amplification manner [8]. VEGF receptors (VEGFR-1 and R-2) are overexpressed in psoriatic skin. Further, VEGF can enhance VEGFR expression in keratinocytes. Transcriptional activation of VEGF and its receptor, VEGFR-1, are mediated by hypoxia-inducible factors (HIFs). Expression of HIF isoforms is strongly upregulated in psoriatic skin, and HIF-1 colocalizes with VEGF in the psoriatic epidermis [9].

In mice, VEGF overexpressed selectively in basal keratinocytes exhibited a chronic skin inflammation with increased numbers of tortuous capillaries and expression of VEGFR-1 and VEGFR-2 [10]. Other studies showed that VEGF transgenic mice whose appearances and immunopathology looked similar to human psoriatic skin [11]. Additionally, overexpression of angiopoietin receptor, Tie2, develops psoriasis-like phenotypes in mice [12]. IL-9 has an angiogenic effect both *in vitro* and* in vivo* [13], and intradermal injection of IL-9 induces VEGF and CD31 overexpression [13]. Other studies also demonstrated that mice models for psoriasis are ameliorated by interfering angiogenesis by recombinant disintegrin domain of ADAM-15 [14] and pigment epithelium-derived factor [15], suggesting that angiogenesis is a possible therapeutic target of psoriasis.

## 3. Angiogenesis of Joint Lesions in PsA

### 3.1. Cellular Infiltrates

Previous studies showed that synovial T-cells in PsA are functionally active, which may migrate also into the psoriatic skin as well as inflamed enthesis. T-cells, cytokines, chemokines, and matrix metalloproteinases (MMPs) are supposed to play important roles in the inflammatory process leading to the destruction of joint tissues. Not only T-cells but also B-cells are seen in the synovium, occasionally forming primitive germinal centers; however the implication of B-cell infiltration is unclear. Abundant T-cells, both CD4+ and CD8+ with clonal or oligoclonal expansions, are infiltrated in the synovial tissues of PsA [16], most of which are activated memory T-cells expressing HLA-DR and CD45RO [17]. T-cells bearing the same T-cell receptor Vβ subsets are present in both joint and skin lesions [18]. Recruitment of T-cells into the synovium may be mediated by chemokines, such as CCL2, CXCL13, CCL21, and CCL22 [19, 20]. CCL22 (macrophage-derived chemokine) was demonstrated to be expressed in the synovial membrane and produced in the synovial fluids of PsA [21]. CCL22 and its ligand CCR4 play an important role in attracting skin-specific memory T-cells to the synovial tissues [2]. T-cell-derived cytokines such as IL-1β, IL-2, IL-10, interferon-*γ* (IFN-*γ*), and TNF-*α* are dominantly detected in the synovium [22, 23]. On the other hand, recent advances indicate that psoriasis is a Th17-mediated inflammatory disease [24]. Th17 cells produce IL-17, which promotes neutrophil migration and proliferates downstream inflammatory molecules. Also, Th17 cells secret IL-6, IL-21, IL-22, TNF-*α*, and IFN-*γ*. IL-23 is their downstream cytokine, promotes the differentiation and growth of IL-17, and upregulates IL-22. IL-20 and IL-22 activate STAT3 in keratinocytes [25]. Thus, the IL-23/IL-17 inflammatory pathway is central to the pathogenesis of psoriasis. IL-23 is overproduced by dendritic cells and keratinocytes. IL-23 activates downstream mediators such as STAT3 and IL-22. Subcutaneous injections of IL-23 in mice induced psoriasis-like changes of erythema and induration as well as histological features such as acanthosis and parakeratosis [26, 27], and this IL-23-induced psoriasis-like inflammation requires CCR-6 [28]. IL-23 transgenic mice show inflammatory cutaneous reaction resembling psoriasis with increased number of T-cells and Langerhans cells [29].

 Infiltration of CD68+ macrophages was less in synovium of PsA as compared with RA, but expression of macrophage-derived cytokines such as TNF-*α*, IL-1, IL-10, and IL-15 was detected in the lining layer and around the vessels [30]. On the contrary, the number of CD163+ macrophages was greater in PsA than in RA [31]. To determine the balance of M1/M2 type macrophages in the PsA synovium, further studies are necessary. Synovial macrophages participate in stimulating angiogenesis.

Neutrophils play an important role in the lesional skin of psoriasis, and also neutrophil infiltration is seen in the synovium. As compared with rheumatoid synovial membranes, infiltration of polymorphonuclear cells is prominent [31].

Dendritic cells (DCs) populations are Langerhans cells, myeloid DCs, and plasmacytoid DCs. Myeloid CD11c+DCs are subdivided into resident and myeloid inflammatory DCs, depending on their expression of CD1c, and in the psoriatic skin, CD11c+CD1c− population is increased in number [32]. Both myeloid DCs and plasmacytoid DCs were present in the synovial fluids of PsA [33, 34]. A large population of these inflammatory DCs express TNF-*α* and inducible nitric oxide synthase (iNOS) and are considered to be equivalent to Tip-DCs. Further, these inflammatory DCs produce IL-20 and IL-23 [35, 36]. Thus, these inflammatory DCs are suggested to play a central role in psoriasis. On the other hand, antimicrobial peptide cathelicidin (LL37) induces IFN-*α* production by plasmacytoid DCs, when complexed with self-DNA. IFN-*α* has been implicated in the induction of psoriasis [37], and inhibition of IFN-*α* prevented the development of psoriatic lesions in mice grafted with human psoriatic skin [38]. IFN-*α* enhances the activation of CD8+ T-cells by antigen-presenting cells [39]. In addition, IFN-*α* amplifies cutaneous inflammation via the induction of chemokines, such as CXCL9, CXCL10, and CXCL11, which recruit their receptor CXCR3 expressing lymphocytes [40], including CD8+ T-cells. Also, plasmacytoid DCs isolated from synovial fluids express CXCR3 and CXCR4 [36], the receptors for CXCL10 and CXCL11, and for CXCL12, respectively. Thus, chemokine-driven recruitment of plasmacytoid DCs into the synovium could be important in the pathogenesis of PsA. Further, cathelicidin stimulates angiogenesis [41].

Mast cells are increased in number in the synovium in PsA as well as enhanced expression of stem cell factor, a mast cell growth factor [42]. Mast cells greatly contribute to angiogenesis, and mast cell degranulation in human induces expression of E-selectin on vascular endothelial cells [43], mast cell-derived TNF-*α* has the potential to induce the early expression of E-selectin [44]. E-selectin was preferentially expressed in the blood vessels in the synovium [42], which suggests that the synovium of PsA is in a continuous state of endothelial activation. Mast cell-derived mediators may also contribute to the synovial hyperplasia and angiogenesis.

### 3.2. Vascular Changes

**Figure 1:**
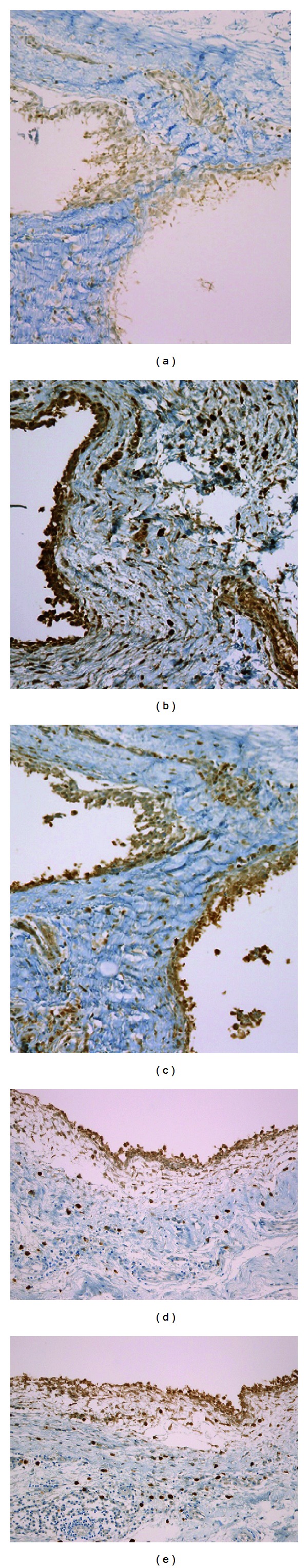
Immunohistological localization of angiogenesis markers (a) VEGF, (b) flt-1, (c) flk-1, (d) angiopoietin, (e) HIF-1*α*) in the PsA synovium. Paraffin-embedded sections were stained by avidin-biotin peroxidase technique.

Synovial angiogenesis is central to synovial proliferation. Angiogenic mediators secreted by synovial tissue cells and/or synovial tissue infiltrating cells are involved in neovascularization. Morphological vascular changes such as tortuous and elongated features are shown by microscopic examination in the blood vessels of synovial membrane of PsA [45]. Angiogenesis is also evident in the psoriatic synovium at early stages and expression of angiogenic cytokines such as VEGF, TGF-β, and angiopoietins [46]. VEGF may be upregulated within the synovium following stimulation with TNF-*α* and IL-1 secreted by synovial cells and also infiltrating cells. Expression of VEGF as well as VEGF receptors (VEGFR-1/Flt1 and VEGFR-2/KDR) is detected in the PsA synovium (Figures 1(a)–1(c)). TNF-*α* is detected in the synovial membrane of PsA, which upregulates angiogenic cytokines [47]. TNF-*α* upregulates the production of proangiogenic VEGF-A splice variants. Anti-TNF therapies reduced VEGF levels in the sera and skin of PsA patients [48, 49], suggesting that TNF-*α* greatly contributes to angiogenesis associated with PsA. Also, TNF-*α* enhances endothelial cells to express adhesion molecules such as intercellular adhesion molecule-1 (ICAM-1), vascular cell adhesion molecule-1 (VCAM-1), and E-selectin [50]. Blood vessels in PsA synovium express a variety of adhesion molecules such as ICAM-1, VCAM-1, and E-selectin [51], which promote leukocyte migration to inflammatory sites. Angiopoietin-1 and -2 and their receptor Tie-2 are involved in angiogenic processes. Angiopoietin-2 is an effector downstream molecule of VEGF signaling pathway and promotes adhesion by sensitizing endothelial cells to TNF-*α* and modulating TNF-*α* induced expression of adhesion molecules on endothelial cells [52]. Recent studies have demonstrated that VEGF and Angiopoietin-2 induced Notch expression, which mediates synovial angiogenesis [53]. In addition to a number of properties described before, IL-17 stimulates endothelial cell migration and cord formation [54] and plays an important role in arthritis. Act1 (nuclear factor-kappa B (NF-*κ*B) activator 1) is an adaptor protein for the IL-17 receptor and modulates downstream mediators of proinflammatory genes [55]. Mast cells are one of the major sources of IL-17 in the synovium [56]. Hypoxia is induced in joint inflammation. Expression of angiopoietin and HIF-1*α* is detected in the PsA synovium (Figures 1(d) and 1(e)).

### 3.3. Synovial Fibroblasts

In the inflamed synovium, synovial fibroblasts are stimulated by proinflammatory cytokines and may change the phenotype which secret inflammatory mediators, matrix-degrading enzymes, and chemotactic molecules. Toll-like receptors (TLRs) play an important role in the regulation of innate and adaptive immune responses. Recent findings demonstrate functionally active TLRs on synovial fibroblasts are involved in the regulation of inflammatory responses in the synovial tissues of RA and spondylarthropathy [57]. Stimulation of TLR-2 pathway in synovial fibroblasts with TNF-*α* induces translocation of NF-*κ*B, which leads to the secretion of proinflammatory cytokines and MMPs [58]. MMP is speculated to play an important role in joint inflammation and tissue destruction. TLRs activate signaling pathways inducing a number of mediators including MMPs [59, 60]. So far, very few data are available on MMPs expression in the synovial tissues of PsA [61]. MMP-1 and MMP-3 are found in the sublining layer cell populations as well as in some lining layer cells, which may be responsible for the severity of the joint symptoms of PsA. Overexpression of dominant-negative forms of MyD 88 significantly downregulated TNF-*α* and also MMPs in cultured synovial membranes isolated from RA patients [62]. Further, TLR-4-deficient mice showed reduced joint inflammation [63]. TLR-2 and TLR-4 may play an important role in the production of MMPs, which contribute to the joint destruction in the synovium. Enhanced expression of TLR-2 and TLR-4 in the PsA synovium has been reported [64]. Increased expression of TLR-2 and -4 have been seen on CD4+ CD28+ T-cells in the peripheral blood of patients with PsA [65]. Expression of TLR-2 and TLR-4 is upregulated by TNF-*α* and IFN-*γ*, as well as IL-1 and lipopolysaccharide (LPS) [66].

### 3.4. Synovial Fluids

CD8+ T-cells are significantly increased with clonal expansions in synovial fluids, but CD4+ T-cells are few [16, 67]. VLA-1 integrin expressing T-cells isolated from synovial fluids display an oligoclonal repertoire [68]. High levels of TNF-*α*, TNF-*α* receptors, IL-1, IL-6, IL-8, IL-13, and other proinflammatory cytokines are found in the synovial fluids, which however are not as much as those contained in RA synovium [69–71]. IL-22 is a downstream effector of IL-23-induced inflammation and epidermal acanthosis in murine models [26]. IL-22 regulates the differentiation and migration of keratinocytes as well as production of antimicrobial proteins from keratinocytes [25, 72]. Thus, IL-22 plays a crucial role in psoriasis. IL-22 levels are significantly elevated in synovial fluids [73]. IL-22 induced significant proliferation of synoviocytes, suggesting that IL-22 contributes to the synovial proliferation in PsA. Interestingly, IL-22 enhances the expression of MMP-1 and MMP-3 [74], suggesting an important role of Th17/Th22 cytokines in the local PsA synovial tissues. CXC chemokines attract neutrophil granulocytes and T-cells. CXCL9 production was significantly high in the synovial fluids of PsA [75], suggesting that CXCL9 is an important chemokine in autoimmune arthritis of PsA. Significant roles of MMP-2 and MMP-9 in angiogenesis have been explored. MMP-9 levels are significantly higher in the synovial fluids of early PsA [76].

## 4. Conclusion

Angiogenic pathways are implicated to play an important role in the pathogenesis of PsA, which drives inflammation, synovial fibroblasts activation, and joint damages. Inhibition of angiogenesis targeting or controlling of angiogenic molecules or angiogenesis-induced cellular events may lead to the novel therapies for PsA.
